# The association between social support and self-rated health in midlife: are men more affected than women?

**DOI:** 10.1590/0102-311XEN106323

**Published:** 2023-12-11

**Authors:** Hisrael Passarelli-Araujo

**Affiliations:** 1 Centro de Desenvolvimento e Planejamento Regional, Universidade Federal de Minas Gerais, Belo Horizonte, Brasil.

**Keywords:** Gender Differences, Social Support, Middle-Aged Adults, Diferenças de Gênero, Apoio Social, Pessoa de Meia-Idade, Diferencias de Género, Apoyo Social, Persona de Mediana Edad

## Abstract

Social support from family and friends is recognized as an important social determinant of health, given its protective effects on individuals’ physical and mental well-being. While most studies have focused on older adults, investigating midlife health is equally crucial since middle-aged individuals are also susceptible to the harmful health outcomes of inadequate social support from friends and family. This study contributes to the debate by examining whether social support is associated with self-rated health among middle-aged Brazilian adults and how this relationship varies between men and women. Using data from the nationwide *Brazilian National Health Survey* conducted in 2019, logistic regression models were employed to assess differences in self-rated health, accounting for confounding factors. The sample comprised 31,926 middle-aged adults, of which 52.5% were women. The overall prevalence of poor self-rated health was 40.7%, with a significant difference between men and women. Results from this study suggest that having no friends or family members to rely on, both during good and challenging times, was associated with poorer self-rated health. However, the strength of this association differs by gender, with social support from friends playing a more critical role in women’s self-rated health. On the other hand, family support was associated with male self-rated health, particularly for men with three or more family members they can rely on. Future studies should consider cultural and contextual factors to better understand other dimensions of social support and its association with midlife health.

## Resumo

O suporte social de familiares e amigos é reconhecido como um importante determinante social da saúde com base em seus efeitos protetores sobre o bem-estar físico e mental dos indivíduos. Embora a maioria das pesquisas tenha se concentrado em adultos mais velhos, investigar a saúde na meia-idade é igualmente crucial, já que estes indivíduos também são suscetíveis aos resultados prejudiciais à saúde decorrentes do suporte social inadequado de amigos e familiares. Este estudo contribui para o debate ao examinar se o suporte social está associado à autoavaliação da saúde entre adultos brasileiros de meia-idade e como essa relação varia entre homens e mulheres. Usando dados da *Pesquisa Nacional de Saúde* realizada em 2019, modelos de regressão logística foram empregados para avaliar diferenças na autoavaliação da saúde, contabilizando fatores de confusão. A amostra foi composta por 31.926 adultos de meia-idade, dos quais 52,5% eram mulheres. A prevalência geral de autoavaliação de saúde ruim foi de 40,7%, com diferença significativa entre homens e mulheres. Os resultados deste estudo sugerem que não ter amigos ou familiares com quem contar em momentos bons ou desafiadores esteve associado à pior autopercepção de saúde. No entanto, a força dessa associação difere de acordo com o gênero, sendo que o suporte social de amigos desempenha um papel mais importante na autoavaliação da saúde das mulheres do que na dos homens. Por outro lado, o apoio familiar esteve associado à autoavaliação da saúde masculina, particularmente para homens com três ou mais membros da família com quem podem contar. Estudos futuros devem considerar fatores culturais e contextuais para compreender melhor outras dimensões do suporte social e sua associação com a saúde na meia-idade.

## Resumen

El apoyo social de la familia y de amigos se reconoce como un importante determinante social de salud basado en sus efectos protectores sobre el bienestar físico y mental de los individuos. Aunque la mayoría de las investigaciones se ha centrado en adultos mayores, investigar la salud en la mediana edad también es esencial, una vez que estos individuos también son susceptibles a los resultados perjudiciales para la salud resultantes de un apoyo social inadecuado de amigos e de la familia. Este estudio contribuye al debate al investigar si el apoyo social está asociado con la autoevaluación de salud entre adultos brasileños de mediana edad y cómo esa relación varía entre hombre y mujeres. Usando datos de la *Encuesta Nacional de Salud* realizada en 2019, se utilizaron modelos de regresión logística para evaluar diferencias en la autoevaluación de salud, contabilizando factores de confusión. La muestra se compuso de 31.926 adultos de mediana edad, de los cuales el 52,5% eran mujeres. La prevalencia general de autoevaluación de mala salud fue del 40,7%, con diferencia significativa entre hombres y mujeres. Los resultados de este estudio sugieren que no tener amigos o familiares en los que confiar en buenos o malos momentos se asoció con la peor autopercepción de salud. Sin embargo, la fuerza de esa asociación es diferente según el género, ya que el apoyo social de amigos es más importante en la autoevaluación de salud de las mujeres que en la autoevaluación de los hombres. Por otro lado, el apoyo familiar se asoció con la autoevaluación de la salud masculina, particularmente para hombres que tenían tres o más personas de la familia en los que confiar. Estudios futuros deben tener en cuenta factores culturales y contextuales para mejor comprender otras dimensiones del apoyo social y su asociación con la salud en la mediana edad.

## Introduction

Social support from family and friends is crucial for maintaining health and well-being. It is a broad concept based on interpersonal interactions, in which individuals perceive they have access to reliable friends or family members to rely on, both during good and challenging times ^1^ ^,^ ^2^. Good social relationships provide emotional and practical resources people need to feel cared for and valued, which can encourage the adoption of healthier behaviors ^3^. For this reason, social support is widely recognized by the scientific community and the World Health Organization (WHO) as an important health determinant, given its protective effects on individuals’ physical and mental well-being ^3^ ^,^ ^4^. It also demonstrates a positive association with health promotion behaviors, quality of life, and self-realization, directly influencing how individuals perceive their health ^5^ ^,^ ^6^.

While most research on health demography and social epidemiology has focused on older adults, investigating midlife health is equally essential for several reasons. From a demographic perspective, middle-aged adults (often in their 40s and 50s) form a substantial and growing segment of populations in many countries, influencing key demographic indicators such as population size, aging trends, and healthcare use ^7^. Often referred to as the “sandwich generation”, a term that describes those middle-aged adults who are effectively pressured between the obligation to care for their aging parents and support their children ^8^ ^,^ ^9^, middle-aged individuals juggle multiple roles, serving as parents, caregivers, and sources of support for both younger and older generations ^10^. The level of social support they receive and perceive can significantly impact their mental and emotional well-being, caregiving abilities, and overall quality of life ^11^.

From a public health perspective, self-rated health and social support have significant implications for health promotion and disease prevention, especially during middle age, a critical period when many chronic diseases emerge ^12^ ^,^ ^13^. Furthermore, social support plays a pivotal role in buffering the effects of stress and adverse life events. Access to adequate social support can provide individuals with emotional and practical resources to cope with stressors and reduce their negative health impacts ^11^.

Studies exploring the potential effects of social support on self-rated health among middle-aged adults dwelling in Brazil and how it varies among men and women are scarce, which is a surprising gap considering the shared concern about the prevalence of loneliness among individuals in recent decades ^14^ ^,^ ^15^ ^,^ ^16^. Men and women in midlife may experience distinct social expectations, roles, and stressors that can influence their self-rated health. Understanding this relationship is crucial to address their specific health needs and promote gender equity in health ^17^.

This study contributes to the current literature by examining whether social support is associated with self-rated health among middle-aged Brazilian adults and how this relationship varies among men and women. By identifying the factors associated with poor self-rated health and possible gender disparities, this study can inform the development of targeted interventions to improve the health of middle-aged Brazilian adults.

## Methods

### Study design and participants

This cross-sectional study relied on data from the *Brazilian National Health Survey* (PNS), a nationwide, population-based survey conducted in 2019 by the Brazilian Ministry of Health and the Brazilian Institute of Geography and Statistics (IBGE). The PNS 2019 aims to describe the health situation and lifestyles of the Brazilian population and is representative of geopolitical macroregions, states, metropolitan areas, and 27 capitals of the Federative Units. The PNS 2019 draws upon a multistage probabilistic sampling design, including individuals aged 15 years old or over, residing in private households, i.e., built for the exclusive purpose of habitation.

The selected sample included 31,296 middle-aged adults (40-59 years old) who answered questions about social support and self-rated health. No ethical approval was needed, as this was an analysis of publicly available data with no personally identifiable information.

### Main outcome measures

Health differences between men and women were analyzed as gender disparities in health. Despite physical and physiological characteristics, such as chromosomal genotype, hormonal levels, and internal and external anatomy playing a role in health differences, this study recognizes that socially constructed roles, behaviors, and expectations associated with being male or female play the most significant role ^18^. Gender encompasses a wide range of non-biological traits, attitudes, and behaviors ^19^. Disparity was used in this context to refer to systematic, avoidable, and unfair inequalities in health and its social determinants, occurring within and between population groups and disproportionately affect vulnerable populations due to inequalities in underlying social, political, and economic institutions ^20^ ^,^ ^21^.

Individual-level self-rated health (dependent variable) was assessed using the following question: “In general, how would you rate your health?”. Answers to this question range from “very good” to “very poor”. This variable was dichotomized considering individuals who rated their health as “good” or “very good” as having “good” self-rated health and individuals who rated their health as “fair”, “poor”, or “very poor” as having “poor” self-rated health.

Information on social support was based on the following variables in the PNS 2019: “How many (family members/relatives or friends) can you count on in good or bad times?”. From this question, social support was defined as the perceived availability and adequacy of emotional, informational, and tangible resources provided by family members/relatives or friends during times of need or stress. The variables on social support in PNS 2019 present four distinct categories: none, one, two, and three or more. Thus, in this study, lack of social support refers to individuals who reported having no family members or friends to rely on. Social support is the main explanatory variable that was hypothesized to link with self-reported health, but other control variables were included as well.

The set of covariates considered in this study encompasses various aspects, including demographic and socioeconomic attributes, health behaviors, and healthcare access, all of which can significantly influence an individual’s self-rated health. To capture the demographic characteristics, age was categorized into four groups: 40-44, 45-49, 50-54, and 55-59 years. Additionally, household location (urban/rural), region of residence (North, Northeast, Central-West, Southeast, and South), marital status, and race/skin color (white, black, mixed-race, and other) were included as relevant factors affecting self-rated health. Socioeconomic attributes were measured by schooling level, which was divided into three categories: low (0-7 years), middle (8-11 years), and high (12 years or more). Moreover, being a current smoker was used as a proxy for health behaviors, as smoking habits can significantly impact overall health and well-being.

To account for physical and mental health status, binary variables were included for chronic diseases (diagnosis of any chronic, physical, mental, or long-term illness), obesity (body mass index - BMI ≥ 30kg/m^2^), and depression diagnosis. The latter was assessed by investigating whether the individual had ever received a diagnosis of depression from a physician or mental health professional (psychiatrist or psychologist). This study also incorporated a dummy variable for health insurance coverage to assess the impact of healthcare access on self-rated health.

### Statistical analysis

After selecting eligible individuals and potential variables for this study, a descriptive analysis was conducted based on the dependent variable and its covariates. Categorical variables were described by their absolute and relative frequency. Pearson’s chi-squared test with Yates’ continuity correction was used for categorical variables when comparing differences between groups in the descriptive analysis. Cramer’s V was employed to measure the association between the nominal variables. P-values above 0.05 were interpreted as insufficient evidence to differentiate groups.

Logistic regression models were employed to test for differences in self-rated health between middle-aged adults. Separate models were estimated for men and women to analyze gender differences in the association. The models were adjusted for potentially confounding variables such as sociodemographic characteristics, adulthood socioeconomic status, health behaviors, and physical and mental health status.

A set of models was generated to test the additive and interactive effects between the variables. Sensitivity and residual analyses were also performed in the preliminary model selection rounds. Odds ratios (OR) - a measure of association that compares the odds of an event occurring in one group to occur in another - were used to present the results. Only the final fitted models were presented in this study. Results were considered significant at p-value < 0.05. All estimations were performed using R program (https://www.r-project.org/) with appropriate methods to handle complex survey designs such as PNS 2019.

## Results

**Figure 1:**
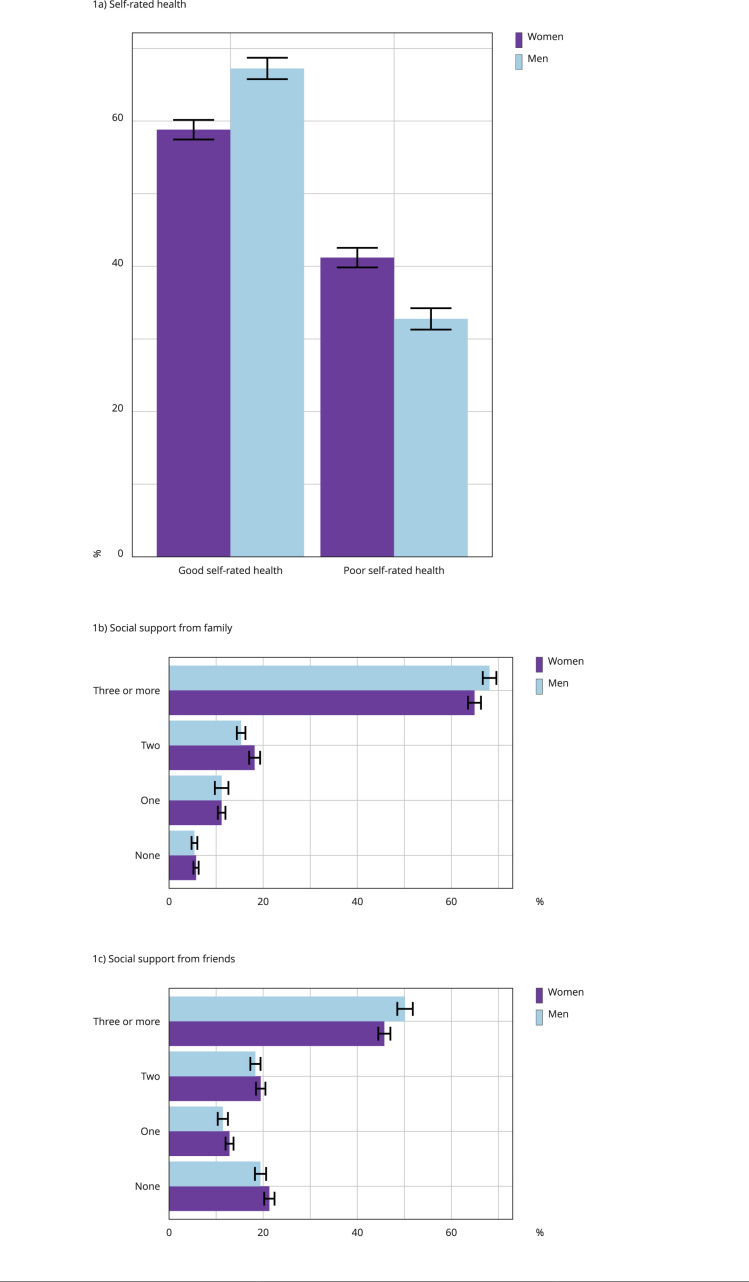
Distribution of middle-aged Brazilian adults selected for the study according to self-rated health, social support from family and from friends, disaggregated by gender. Brazil, 2019.

Figure 1 illustrates the proportion of individuals based on self-rated health (good and poor) (Figure 1a), social support received from family members (Figure 1b), and social support received from friends (Figure 1c) for both men and women, with 95% confidence intervals (95%CI). The overall prevalence of poor self-rated health among middle-aged Brazilians was 40.7%, with a significant difference between men (32.7%, 95%CI: 31.3; 34.2) and women (41.2%, 95%CI: 39.8; 42.5), suggesting a higher prevalence of women reporting poor self-rated health compared to men.

Approximately 5.7% of women and 5.3% of men reported not receiving any social support from family members (Figure 1b). More than 65% of the sample reported receiving family support from three or more members, with a higher proportion of men in this category. Regarding social support from friends, 21.4% of women reported having no friends to rely on in good or bad times. For men, this value was slightly lower, around 19.5%. Moreover, concerning social support from friends, a higher proportion of men have three or more friends to rely on compared to women (Figure 1c).

Table 1 shows other sample characteristics stratified by gender. Regarding schooling level, approximately 20% (n = 3,763) of women had a high education level compared to 17% (n = 2,576) of men. Most women in the sample presented cases of chronic, physical, mental, or long-term illness (65.1%) compared to men (48.7%). Furthermore, the prevalence of depression was greater among women (16.7%) than men (5.3%). The sample population predominantly resided in urban areas (77.4%), with most participants concentrated in the Northeast (34.2%) and Southeast (22.3%) regions. Most participants self-declared as mixed-race (50.2%). Smoking habit is more prevalent among men (17.5%) compared to women (12.2%) in the sample. There is a higher proportion of women with obesity (25.4%) compared to men (21.6%). Additionally, the proportion of women with health insurance (24%) also surpasses that of men (21.7%).

**Table 1:** Description of the study variables by men and women (n = 31,926). *Brazilian National Health Survey*, 2019 (PNS 2019).

	Total	Women	Men	p-value
	n (%)	n (%)	n (%)	
Overall	31,926 (100.0)	16,763 (52.5)	15,163 (47.5)	
Age group (years)				< 0.001
40-44	8,526 (26.7)	4,485 (26.8)	4,041 (26.7)	
45-59	7,922 (24.8)	4,101 (24.5)	3,821 (25.2)	
50-54	7,809 (24.5)	4,112 (24.5)	3,697 (24.4)	
55-59	7,669 (24.0)	4,065 (24.2)	3,604 (23.8)	
Household location				< 0.001
Urban	24,724 (77.4)	13,725 (81.9)	10,999 (72.5)	
Rural	7,202 (22.6)	3,038 (18.1)	4,164 (27.5)	
Region of residence				0.0012
North	5,854 (18.3)	2,946 (17.6)	2,908 (19.2)	
Northeast	10,933 (34.2)	5,863 (35.0)	5,070 (33.4)	
Central-West	3,858 (12.1)	2,047 (12.2)	1,811 (11.9)	
Southeast	7,130 (22.3)	3,757 (22.4)	3,373 (22.2)	
South	4,151 (13.0)	2,150 (12.8)	2,001 (13.2)	
Schooling level				< 0.001
Low	16,139 (50.6)	7,831 (46.7)	8,308 (54.8)	
Middle	9,448 (29.6)	5,169 (30.8)	4,279 (28.2)	
High	6,339 (19.9)	3,763 (22.4)	2,576 (17.0)	
Race/Skin color				0.0128
White	11,718 (36.7)	6,200 (37.0)	5,518 (36.4)	
Black	3,737 (11.7)	1,871 (11.2)	1,866 (12.3)	
Mixed-race	16,019 (50.2)	8,445 (50.4)	7,574 (50.0)	
Other	452 (1.4)	247 (1.5)	205 (1.4)	
Disease diagnosis				< 0.001
Yes	18,302 (57.3)	10,921 (65.1)	7,381 (48.7)	
No	13,624 (42.7)	5,842 (34.9)	7,782 (51.3)	
Depression				< 0.001
Yes	3,598 (11.3)	2,793 (16.7)	805 (5.3)	
No	28,328 (88.7)	13,970 (83.3)	14,358 (94.7)	
Smoking habits				< 0.001
Yes	4,707 (14.7)	2,053 (12.2)	2,654 (17.5)	
No	27,219 (85.3)	14,710 (87.8)	12,509 (82.5)	
Obesity				< 0.001
Yes	7,543 (23.6)	4,264 (25.4)	3,279 (21.6)	
No	24,383 (76.4)	12,499 (74.6)	11,884 (78.4)	
Health insurance				< 0.001
Yes	7,304 (22.9)	4,021 (24.0)	3,283 (21.7)	
No	24,622 (77.1)	12,742 (76.0)	11,880 (78.3)	

Note: p-value of statistical significance for the differences between genders.

Table 2 presents the results of the logistic regression for the overall population and stratified by gender. Results from the overall model showed that men are 17.6% less likely to report poor self-rated health than women, whereas other factors were equal (OR = 0.824, 95%CI: 0.754; 0.900). Social support was also associated with lower odds of reporting poor self-related health. For example, middle-aged adults with two friends are 16.3% less likely to report poor self-rated health (OR = 0.837, 95%CI: 0.737; 0.952) than those without friends. Those who receive support from three or more friends have an even lower chance of reporting poor self-related health, with a 24.8% lower risk than individuals who receive no support from friends.

**Table 2:** Risk of presenting poor self-rated health among middle-aged men and women by selected covariates (n = 31,926). *Brazilian National Health Survey*, 2019 (PNS 2019).

	General		Men		Women	
	OR (95%CI)	p-value	OR (95%CI)	p-value	OR (95%CI)	p-value
Gender						
Women	1.000	-	1.000	-	1.000	-
Men	0.824 (0.754; 0.900)	< 0.001	-	-	-	-
Support from friends						
None	1.000	-	1.000	-	1.000	-
One	1.005 (0.867; 1.165)	0.949	0.992 (0.788; 1.248)	0.944	1.012 (0.832; 1.232)	0.904
Two	0.837 (0.737; 0.952)	0.007	0.823 (0.664; 1.019)	0.075	0.855 (0.715; 1.021)	0.084
Three or more	0.752 (0.667; 0.848)	< 0.001	0.778 (0.643; 0.941)	0.010	0.731 (0.624; 0.858)	< 0.001
Support from family						
None	1.000	-	1.000	-	1.000	-
One	1.095 (0.895; 1.338)	0.378	1.023 (0.781; 1.340)	0.870	1.166 (0.876; 1.553)	0.293
Two	0.983 (0.811; 1.193)	0.866	0.947 (0.737; 1.215)	0.667	1.006 (0.754; 1.341)	0.969
Three or more	0.835 (0.705; 0.989)	0.037	0.755 (0.603; 0.945)	0.014	0.902 (0.710; 1.146)	0.398
Age group (years)						
40-44	1.000	-	1.000	-	1.000	-
45-59	1.186 (1.054; 1.334)	0.005	1.357 (1.113; 1.653)	0.003	1.073 (0.924; 1.246)	0.358
50-54	1.507 (1.348; 1.684)	< 0.001	1.679 (1.416; 1.991)	< 0.001	1.399 (1.191; 1.643)	< 0.001
55-59	1.487 (1.317; 1.679)	< 0.001	1.675 (1.408; 1.993)	< 0.001	1.371 (1.17; 1.606)	< 0.001
Household location						
Urban	1.000	-	1.000	-	1.000	-
Rural	1.295 (1.176; 1.426)	< 0.001	1.217 (1.064; 1.393)	0.004	1.374 (1.197; 1.576)	< 0.001
Region of residence						
North	1.000	-	1.000	-	1.000	-
Northeast	1.034 (0.930; 1.150)	0.540	1.055 (0.894; 1.246)	0.527	1.014 (0.870; 1.182)	0.860
Central-West	0.617 (0.535; 0.711)	< 0.001	0.669 (0.534; 0.838)	< 0.001	0.572 (0.474; 0.689)	< 0.001
Southeast	0.510 (0.453; 0.574)	< 0.001	0.530 (0.439; 0.640)	< 0.001	0.486 (0.410; 0.577)	< 0.001
South	0.460 (0.399; 0.530)	< 0.001	0.576 (0.467; 0.711)	< 0.001	0.375 (0.304; 0.462)	< 0.001
Schooling level						
Low	1.000	-	1.000	-	1.000	-
Middle	0.650 (0.586; 0.720)	< 0.001	0.580 (0.499; 0.675)	< 0.001	0.707 (0.610; 0.818)	< 0.001
High	0.330 (0.288; 0.380)	< 0.001	0.330 (0.265; 0.410)	< 0.001	0.332 (0.276; 0.399)	< 0.001
Race/Skin color						
White	1.000	-	1.000	-	1.000	-
Black	1.307 (1.130; 1.512)	< 0.001	1.191 (0.957; 1.482)	0.117	1.407 (1.170; 1.691)	< 0.001
Mixed-race	1.337 (1.218; 1.467)	< 0.001	1.359 (1.188; 1.555)	< 0.001	1.301 (1.148; 1.473)	< 0.001
Other	1.113 (0.796; 1.554)	0.532	1.023 (0.635; 1.647)	0.926	1.204 (0.760; 1.908)	0.429
Disease diagnosis						
Yes	1.000	-	1.000	-	1.000	-
No	0.271 (0.245; 0.300)	< 0.001	0.252 (0.218; 0.292)	< 0.001	0.295 (0.259; 0.334)	< 0.001
Depression						
Yes	1.000	-	1.000	-	1.000	-
No	0.519 (0.459; 0.587)	< 0.001	0.531 (0.409; 0.689)	< 0.001	0.500 (0.427; 0.584)	< 0.001
Smoking						
Yes	1.000	-	1.000	-	1.000	-
No	0.846 (0.756; 0.946)	0.003	0.831 (0.711; 0.971)	0.020	0.857 (0.725; 1.012)	0.070
Obesity						
Yes	1.000	-	1.000	-	1.000	-
No	0.680 (0.616; 0.752)	< 0.001	0.797 (0.692; 0.917)	0.002	0.604 (0.532; 0.686)	< 0.001
Health insurance						
Yes	1.000	-	1.000	-	1.000	-
No	1.767 (1.553; 2.010)	< 0.001	1.727 (1.444; 2.064)	< 0.001	1.776 (1.503; 2.098)	< 0.001

95%CI: 95% confidence interval; OR: odds ratio.

Regarding family support received from family/relatives, individuals who reported receiving support from three or more relatives were 16.5% less likely to report poor self-related health than those who did not receive any support. However, there was insufficient evidence to establish differences in self-rated health between individuals with only one or two family members compared to the base group at a 95%CI.

Factors associated with a greater chance of reporting poor self-related health also included age, such as those in the 55-59 age group (OR = 1.487, 95%CI: 1.317; 1.679), living in rural areas (OR = 1.295, 95%CI: 1.176; 1.426), residing in the North or Northeast regions (OR = 1.034, 95%CI: 0.930; 1.150), having low schooling level, and being black (OR = 1.307, 95%CI: 1.130; 1.512) or being mixed-race (OR = 1.337, 95%CI: 1.218; 1.467). Marital status was not associated with poor self-rated health among middle-aged Brazilian adults.

Regarding health characteristics, individuals diagnosed with a physical or mental illness were 72.9% more likely to report poor self-related health than those without any diagnosed disease. Additionally, those who have not been diagnosed with depression (OR = 0.519, 95%CI: 0.459; 0.587), did not smoke (OR = 0.846, 95%CI: 0.756; 0.946), and had no obesity (OR = 0.680, 95%CI: 0.616; 0.752) were at lower risk of reporting poor self-realted health.

Results from models stratified by men and women revealed interesting gender disparities in the association between social support and self-rated health (Table 2). The results showed that social support received from friends is a more significant factor for women’s self-rated health than men’s. Specifically, women with three or more friends are 26.9% less likely to report poor health than their counterparts without friends (OR = 0.731, 95%CI: 0.624; 0.858). On the other hand, men with three or more friends are 22.2% less likely to report poor health than men without friends (OR = 0.778, 95%CI: 0.643; 0.941). These differences are significant compared to the reference group without friends (p-value = 0.01 for men and p-value < 0.001 for women). However, it was not possible to establish differences in self-rated health between individuals with only one or two friends compared to the baseline group at a 95%CI.

Regarding family support, the results suggest a weaker association with self-rated health for both men and women compared with social support received from friends, except for men with three or more family members they can rely on (Table 2). In this case, having three or more family members/relatives that men can count on in good or bad times is associated with a 24.5% lower chance of reporting poor health than men without family support (OR = 0.755, 95%CI: 0.603; 0.945). However, in the case of women, the gender-separated logistic regression model did not provide enough statistical evidence to establish an association between family support and poor self-rated health at a 95%CI.

The variables related to sociodemographic characteristics, adulthood socioeconomic status, health behaviors, and physical and mental health status for men and women showed a consistent pattern with the general model. Specifically, poor self-rated health for men and women was associated with residing in rural households, living in the North or Northeast regions, having low education, being black or mixed-race, having a disease diagnosis, suffering from depression, smoking habit (women), having obesity, and lacking health insurance.

## Discussion

This study investigated whether the lack of social support was associated with poor self-rated health among middle-aged Brazilian adults and how it varied among men and women. The results revealed several findings that shed light on the importance of social support in shaping self-rated health outcomes. After adjusting for potential confounders, this study showed that having no friends or family to rely on in good or challenging times was associated with poorer self-rated health.

Our findings also suggest that gender differences significantly affect self-rated health among middle-aged Brazilian adults. Specifically, men were less likely to report poor self-rated health than women, with a 17.6% lower likelihood when controlling for other factors. This gender disparity in self-rated health aligns with the so-called “gender paradox”, which refers to the observation that although women tend to have a higher life expectancy and lower mortality rates than men, they tend to report poorer self-rated health and experience more chronic health conditions than men ^22^.

The fact that women often tend to rate their health lower than men can be attributed to a combination of social and cultural factors. In the social dimension, one possible explanation is that women may have a higher awareness of their own health status than men, and therefore may be more likely to report poor health ^23^. When considering the “sandwich generation” concept, in which middle-aged adults are responsible for caring for aging parents and supporting their children, the current literature suggest that women are more affected than men due to societal norms placing a greater caregiving burden on them ^24^. This situation leads to increased stress and challenges in balancing work and family responsibilities, impacting women’s self-rated health and resulting in lower health ratings compared to men ^25^.

Women may also be more willing to seek medical attention and report symptoms, leading to a higher likelihood of a diagnosis of chronic health conditions ^26^. Conversely, men may be more likely to deny or downplay health problems, leading to underreporting of poor health ^27^. Moreover, men may be less likely to seek or receive emotional support from their social networks due to cultural norms that encourage them to be self-reliant and independent ^28^. These interconnections between work-life balance, the sandwich generation phenomenon, and cultural norms can collectively contribute to the observed gender disparities in self-rated health among middle-aged Brazilian adults.

Despite the widely diffused idea of a gender paradox in the literature, the debate surrounding this concept has been inconclusive. While some studies propose that men and women differ significantly in their self-related health evaluations due to the influence of various biological, social, and cultural factors, other studies suggest that men and women may be more similar in how they incorporate a wide range of chronic and acute health conditions, functioning, healthcare use, and health behaviors in their self-rated health evaluation ^29^. Our study, on the other hand, diverges from this previous idea, given that there are marked differences between self-rated health of Brazilian men and women, even controlling for chronic conditions, health behaviors, and socioeconomic status, as also observed in other settings ^30^. Such discussion highlights the complexity of the relationship between gender and self-rated health.

Regarding social support, the results demonstrated that a substantial proportion of middle-aged Brazilian adults receive support from family and friends. However, gender differences were visible in the patterns of social support received. The logistic regression analyses revealed that social support from both friends and family members was associated with better self-rated health among middle-aged adults. These findings align with the social support literature, indicating that strong social networks and interpersonal relationships positively impact individuals’ self-rated health ^31^.

The observed gender disparities in the association between social support and self-rated health are particularly intriguing. The results suggest that social support from three or more friends shows a more significant impact on women’s self-rated health compared to men. Women with three or more friends were 26.9% less likely to report poor health, whereas for men, the reduction in the odds of reporting poor health was 22.2%. This finding could be explained by gender differences in coping mechanisms and the tendency of women in maintaining closer relationships with their friends, placing more importance on social support received from them ^4^. The results are consistent with previous research suggesting that social support from friends is a strong predictor of health outcomes ^32^.

Conversely, family support seems to play a minor role in shaping women’s self-rated health compared to social support from friends. For men, having three or more family members they can rely on was associated with a 24.5% lower chance of reporting poor health. This result aligns with previous research demonstrating the importance of family support in promoting men’s health and well-being ^27^. Receiving support from three or more family members may be particularly important for Brazilian men, possibly due to cultural norms that place greater emphasis on family relationships and support ^33^.

Although no significant association between family support and poor self-rated health was found for women, such outcome must be cautiously interpreted. The complexity of women’s social networks and the influence of varying cultural norms regarding the role of family support in their lives may underlie these findings. For instance, women may rely more on external support systems beyond immediate family members ^34^, such as friends or community networks, which could contribute to the muted impact of family support on their self-rated health. Additionally, societal expectations of women as caregivers may lead to potential underreporting of health issues, possibly masking the true relationship between family support and self-rated health among women ^35^. To achieve a deeper understanding of these gender-specific patterns, further research should explore the underlying mechanisms and cultural dynamics that could be driving the observed association between family support and self-rated health among women.

This study contributes to the literature on social support and self-rated health by investigating gender differences in the association between social support and self-rated health among middle-aged Brazilian adults. However, some limitations should be considered. First, the study cross-sectional design does not allow us to establish causality or temporal relationships between social support and self-rated health. Longitudinal studies are needed to investigate the directionality of the association between these variables. Second, the study relies on self-reported social support and self-rated health measures, which may be subject to bias. Self-rated health may be subject to varying perceptions based on individual characteristics such as culture, age, and gender. Future research could benefit from incorporating objective health measures to validate self-rated health assessments, as proposed by Lazarevič ^36^. Similarly, the perception of social support can also be influenced by several factors, including the quality and closeness of interpersonal relationships, the availability and accessibility of social resources, and the person’s ability to seek and use available support. Future studies should also consider the influence of cultural and contextual factors on the association between social support and self-rated health. This line of investigation should expand beyond the scope of our study, addressing other Latin American countries.

## Conclusion 

In conclusion, this study provides evidence into the association between social support and self-rated health among middle-aged Brazilian adults, with a specific focus on understanding gender disparities in this relationship. Our findings demonstrate that both friends and family social support are linked to better self-rated health in middle-aged adults. Particularly, social support from three or more friends presents a more pronounced impact on women’s self-rated health compared to men, whereas family support plays a more significant role in promoting men’s health. Our study contributes to the ongoing discussion about the impact of social support on health and emphasizes the importance of further research to explore the underlying mechanisms shaping gender differences and other aspects of the association between social support and midlife health.
